# L-arginine conjugates of bile acids-a possible treatment for non-alcoholic fatty liver disease

**DOI:** 10.1186/1476-511X-13-69

**Published:** 2014-04-22

**Authors:** Irina Voloshin, Michal Hahn-Obercyger, Sarit Anavi, Oren Tirosh

**Affiliations:** 1 Institute of Biochemistry, Food Science and Nutrition, The Robert H. Smith Faculty of Agriculture, Food and Environment, The Hebrew University of Jerusalem, PO Box 12, Rehovot 76100, Israel

**Keywords:** Liver damage, Steatosis, Over-nutrition, Obesity, Metabolic syndrome

## Abstract

**Background:**

Non-alcoholic fatty liver disease (NAFLD) is a continuum of diseases that include simple steatosis and non-alcoholic steatohepatitis (NASH) ultimately leading to cirrhosis, hepatocellular carcinoma and end stage liver failure. Currently there is no approved treatment for NASH. It is known that bile acids not only have physiological roles in lipid digestion but also have strong hormonal properties. We have synthesized a novel chenodeoxycholyl-arginine ethyl ester conjugate (CDCArg) for the treatment of NAFLD.

**Methods:**

Chemical synthesis of CDCArg was performed. Experiments for prevention and treatment of NAFLD were carried out on C57BL/6 J male mice that were treated with high fat diet (HFD, 60% calories from fat). CDCArg or cholic acid bile acids were admixture into the diets. Food consumption, weight gain, liver histology, intraperitoneal glucose tolerance test, biochemical analysis and blood parameters were assessed at the end of the experiment after 5 weeks of diet (prevention study) or after 14 weeks of diet (treatment study). In the treatment study CDCArg was admixture into the diet at weeks 10–14.

**Results:**

In comparison to HFD treated mice, mice treated with HFD supplemented with CDCArg, showed reduced liver steatosis, reduced body weight and decreased testicular fat and liver tissue mass. Blood glucose, cholesterol, insulin and leptin levels were also lower in this group. No evidence of toxicity of CDCArg was recorded. In fact, liver injury, as evaluated using plasma hepatic enzyme levels, was low in mice treated with HFD and CDCArg when compared to mice treated with HFD and cholic acid.

**Conclusion:**

CDCArg supplementation protected the liver against HFD-induced NAFLD without any toxic effects. These results indicate that basic amino acids e.g., L-arginine and bile acids conjugates may be a potential therapy for NAFLD.

## Background

Non-alcoholic fatty liver disease (NAFLD) is a continuum of diseases that include simple steatosis and non-alcoholic steatohepatitis (NASH) ultimately leading to cirrhosis, hepatocellular carcinoma (HCC) and end stage liver failure which develop in the absence of excessive alcohol intake [1-3]. NAFLD affects 30% of the general population and 70-80% of diabetic and obese patients [4-6]. Risk factors of the disease include low physical activity, dietary factors, gut microbiota genetic factors, oxidative stress and sleep apnea [7], fructose enriched beverages [8] etc.

Simple steatosis is considered to have a benign hepatic pathological prognosis. In contrast, NASH is pathologically characterized by the presence of steatosis, inflammation and liver fibrosis and is related to increased mortality. NASH is associated with liver related complications, and it is a leading cause of liver transplantation [9].

Currently there is no approved treatment for NASH. It has been known that bile acids have broad and powerful hormonal properties as gene regulators as well as established physiologic roles in digestion of cholesterol and other lipids. These molecules participate in food digestion as well as in energy homeostasis owing to their physical properties in the enterohepatic circulation. Bile acids may also act as hormones and therefore can influence transcription and activate several signaling pathways [10].

Bile acids are endogenous ligands of the farnesoid X receptor (FXR) and G protein-coupled bile acid receptor (TGR5). Functional studies have demonstrated that both FXR and TGR5 play important roles in regulating lipid and carbohydrate metabolism and inflammatory responses. Importantly, activation of FXR or TGR5 lowers hepatic triglyceride levels and inhibits inflammation. These properties of FXR or TGR5 have indicated that these two bile acid receptors are possible targets for treatment of NAFLD [11].

Despite of the potential protective effect of bile acids against NAFLD due to their signaling role, it is important to note that in animal models of NASH, bile acids act to promote and to exacerbate liver disease. For example, atherogenic diet that contains 0.5 percent of cholic acid (CA) induces oxidative stress and steatohepatitis with hepatocytes ballooning accompanied by insulin resistance, and down-regulates antioxidant genes. Thereby, bile acids further aggravate steatohepatitis [12].

Previously it has been suggested that bile acids aggravate the disease of NAFLD also in humans. Increased bile acids levels, Deoxycholic, Chenodeoxycholic, and CA were elevated in liver tissue of steatohepatitis patients [13]. Another study analysed liver biopsies and serum samples of 113 morbidly obese patients undergoing bariatric surgery, healthy individuals, and moderately obese NAFLD patients. In this study serum bile acids levels were increased in NASH versus simple steatosis and the NAFLD activity score (NAS) correlated with bile acid levels. In addition, the bile acids transporter high-affinity Na^+^ /taurocholate cotransporter polypeptide (NTCP) and the bile acids synthesizing enzyme cholesterol 7 alpha-hydroxylase (CYP7A1) were significantly up-regulated in obese patients. Up-regulation of NTCP and CYP7A1 indicate failure to activate small heterodimer partner (SHP) upon FXR stimulation by increased bile acids concentrations. Therefore, bile acids synthesis and serum bile acids levels correlate with disease severity in NAFLD [14].

In 1975 Yosef and Fisher [15] reported the presence of L-arginocholate in isolated perfused rat liver. The structure was confirmed by mass spectrometry analysis. Regular bile acids, as was analyzed in gallbladder, common ducts and duodenum are conjugated to glycine and taurine. The role of the lesser known basic amino acid bile acid conjugates is intriguing. One possibility is that these bile acids may serve as antagonists to the effect of the regular bile acids. The aim of this study was to develop a novel bile acid molecule that can be used to treat NAFLD and NASH. For that purpose an L-Arginine ethyl ester and chenodeoxycholic acid conjugate (CDCArg) was generated and tested in nutritional models of NASH.

## Results

### Prevention study

#### *Administration of CDCArg induces food consumption and reduced body weight*

**Figure 1:**
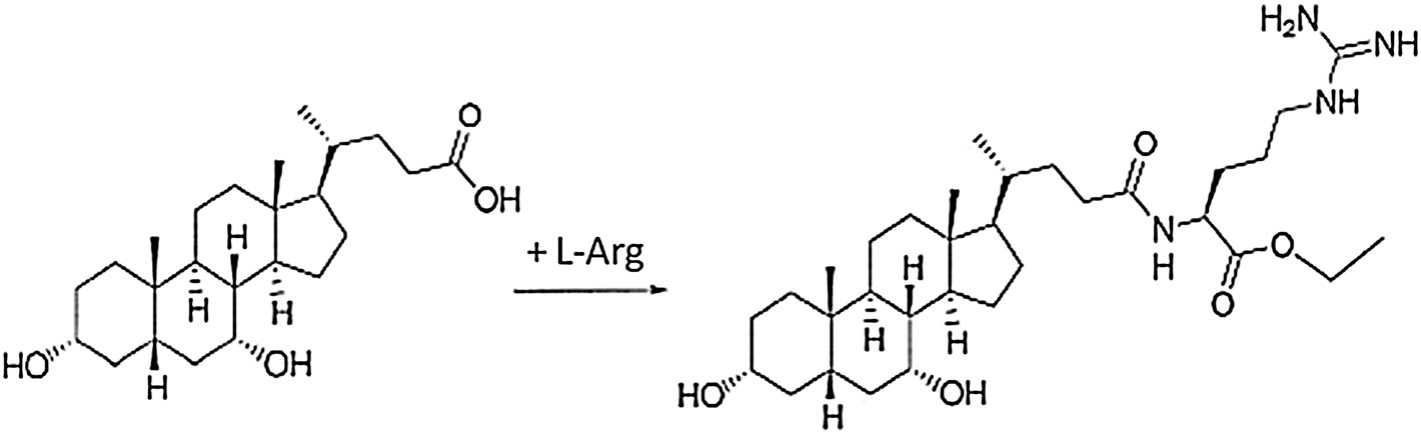
**Chemical stucture of Chenodeoxycholyl–arginine-ethyl ester CDCArg and diagram of synthesis.** The synthetic pathway: Chenodeoxycholic acid (78.5 gm, 0.2 mol) was dissolved in DMF (250 mL) and tributylamine (95.3 mL) in 1000 ml round bottom flask. Ethyl chloroformate (20.16 mL) was slowly added to the mixture at 0°C. The mixture was stirred at 0°C for 30 min. In 500 ml round bottom flask, a mixture of arginine ethyl ester dihydrochloride (60.28 gm, 0.22 mol) and tributylamine (95.3 mL) in DMF (200 mL) was prepared. It was transferred to a dropping funnel and slowly added to the mixture of mixed anhydride prepared from chenodeoxycholic acid and ethyl chloroformate at 0°C (step 1). The mixture was stirred at room temperature overnight. The mixture was poured on ice and the gummy residue separated out was collected by separating from water (77 gm). The water from step 5 was concentrated in a rotary evaporator.The residue obtained after concentration was loaded above a silica bed (300 gm) and eluted with methylene chloride followed by methanol –methylene chloride (1:4). The earlier fractions with 100% methylene chloride were discarded since they mostly contained impurities and solvents (i.e. DMF and tri-buylamine). The later fractions were concentrated and provided a gummy residue (45 gm). The crude product from step 7 (77 gm) and step 11 (45 gm) was dissolved in methanol and combined. It was then purified on the Teledyne Isco 330 gm silica gel column using 10-15% methanol –methylene chloride. The purification was carried out on Combi-Flash using the ELSD detector. Three separate runs were performed. The pure fraction were collected and concentrated on rotary evaporator to give compound 1 (50.8 gm). Reaction was repeated following the above procedure.

**Figure 2:**
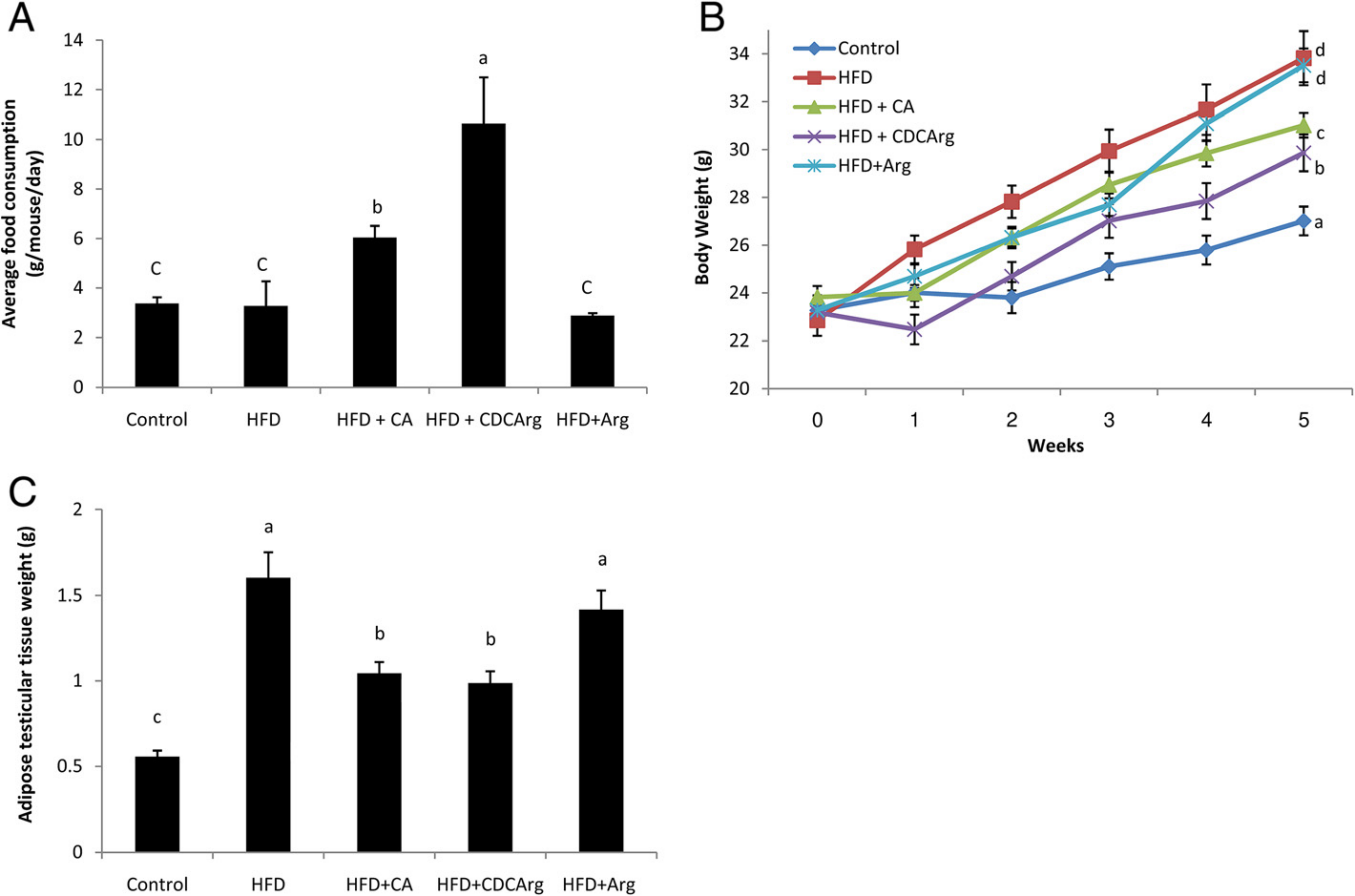
**Effect of CDCArg on body weight, food intake and adipose tissue weight.** Mice were fed (Ad libitum) for 5 weeks. Treatment groups: Control - low fat diet (LFD), high fat diet (HFD), CDCArg (0.5% w/w) added to the HFD, HFD + cholic acid (CA, 0.5% w/w) and HFD + L-arg 1.25% in the drinking water, n = 8-9 in each group. **(A)** Daily consumption of food at weeks 2, 3, 4 and 5. **(B)** Animals weight: Animals weight was measured throughout the experiment at the beginning of each week. **(C)** Testicular adipose tissue weight. Statistics were performed using analysis of repeated measurements of weight. Different groups that are statistically different with probability of *P* < 0.05 are indicated by different letters.

Mice were fed the following diets for five weeks: low fat diet (LFD, control), high fat diet (HFD), and HFD with CA, CDCArg (Figure 1) or L-arginine Although diets were given ab-libitum, food consumption of the HFD + CDCArg group was significantly higher than all other treatments, including the HFD group (Figure 2A).

However, surprisingly, CDCArg significantly inhibited weight gain in HFD + CDCArg group compared with HFD treatment. In fact, HFD + CDCArg-treated mice had the lowest body weight compared to all the other groups with the exception of the LFD control group (Figure 2B). Interestingly, the combination of a different primary bile acid, CA, together with the HFD resulted in similar effects of increased appetite and decreased body weight as of CDCArg. Moreover, adipose tissue mass (testicular fat) was also significantly lower in HFD + CDCArg group compared with HFD group (Figure 2C).

### Effect of the compound CDCArg on blood glucose, adipose tissue weight and plasma insulin & leptin levels

**Figure 3:**
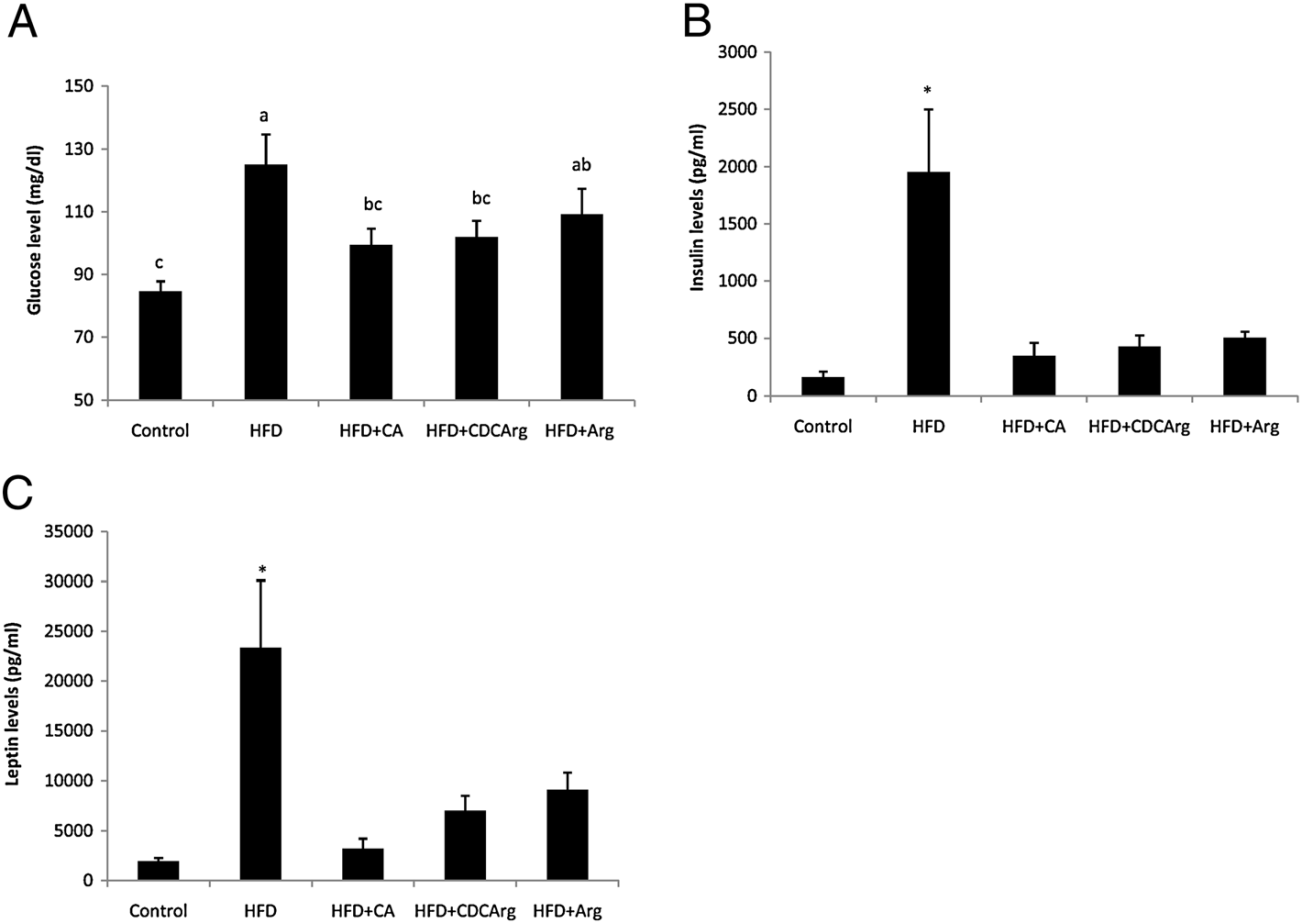
**Effect of the compound CDCArg on blood glucose levels, serum insulin and leptin levels.** Mice were fed (ad libitum) for 5 weeks with LFD, HFD, HFD + CA, HFD + CDCArg and HFD + Arg. **(A)** Blood glucose levels, **(B)** Serum insulin or **(C)** leptin levels. Values are expressed as mean ± SEM (n = 8-9 animals per group). Means without a common letter are statistically different. Means with *are statistically higher compared to all other groups, p < 0.05.

Blood glucose levels were significantly lower in HFD + CDCArg group compared with HFD treated group (Figure 3A). Similarly, plasma insulin and leptin levels were also significantly lower in HFD + CDCArg group compared with the HFD group (Figure 3B,C). Consistent with the above mentioned results, in these experiments the addition of CA or large dose of free L-arginine (around 3 g/kg bw) to the HFD also had positive effects much similar to CDCArg.

### Role of CDCArg in preventing liver damage

**Figure 4:**
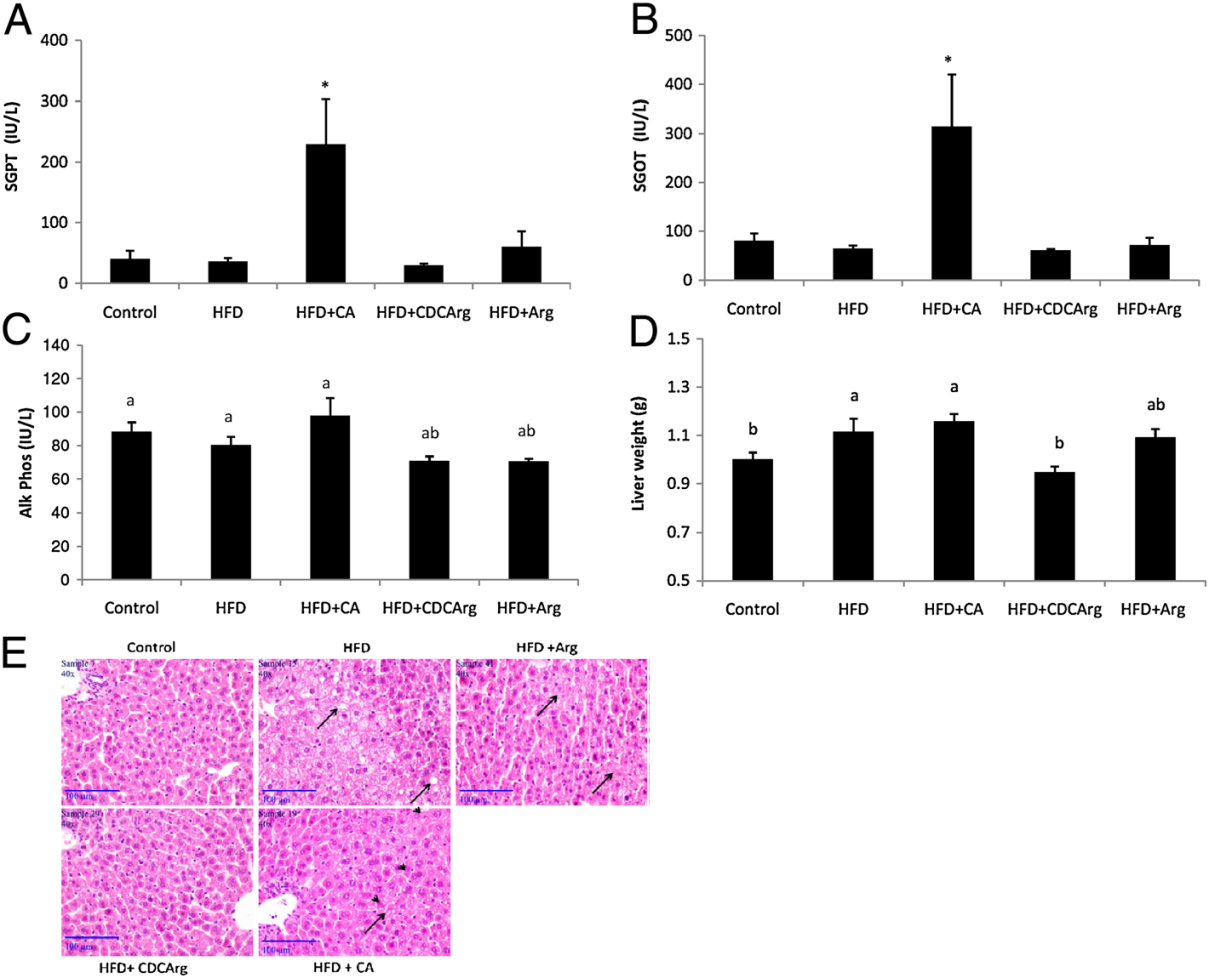
**Effect of CDCArg on hepatic enzyme levels in the serum, liver weight and liver histology.** Mice were fed (ad libitum) for 5 weeks with LFD, HFD, HFD + CA, HFD + CDCArg and HFD + Arg. **(A-C)** Serum SGPT, SGOT or Alkaline Phosphatase levels. **(D)** Liver weight. **(E)** Representative liver histological sections stained with H&E: (magnification 400X). Arrows indicate hepatocytes steatosis and Arrow heads indicate hepatocytes damage (necrosis or apoptosis). Values are expressed as mean ± SEM (n = 8-9 animals per group). Means with different letters are statistically different. Means with *are statistically higher compare to all other groups, p < 0.05.

Hepatic enzyme levels in plasma were measured as an indication of liver damage. Serum glutamic-pyruvic transaminase (SGPT) and serum glutamic oxaloacetic transaminase (SGOT) levels were significantly higher in HFD + CA group compared with all other groups (Figure 4A,B). Conversely, plasma alkaline phosphatase levels were not significantly elevated (Figure 4C). These results indicate that CA is primarily toxic to hepatocytes and to a considerably lesser extent to cholingiocytes in the liver, while CDCArg is not toxic to either cell type.

Hepatomegaly (enlarged livers) can results from several causes including infection/inflammation and metabolic disorders. Indeed, accumulation of lipids in the liver can lead to increased liver weight. Liver weight was substantially lower in HFD + CDCArg group compared with the HFD and HFD + CA groups (Figure 4D). Histological evaluation by H&E staining showed elevated steatosis (score of 3) in the HFD group, while in the HFD + CDCArg group liver steatosis did not differ from that of the control group (Figure 4E).

In comparison to the histological features of CA + HFD treated mice, CDCArg + HFD treated mice showed major differences regarding parameters related to liver damage and hepatocyte injury. In HFD + CA treated mice massive hepatocytes necrosis was observed which correlated with elevated levels of blood liver enzymes AST (SGOT) and ALT (SGPT) (Figure 4E). Conversely, in HFD + CDCArg treated mice, no evidence of liver injury was found and their phenotype did not differ from the control group. To exclude the possibility that these results may be the outcome of general toxicity of the compounds, additional parameters (plasma creatinine, sodium, potassium and chloride levels) were evaluated. No significant differences among groups were observed (Additional file 1: Figure S1).

### Effect of the compound CDCArg on plasma lipid profiles

**Figure 5:**
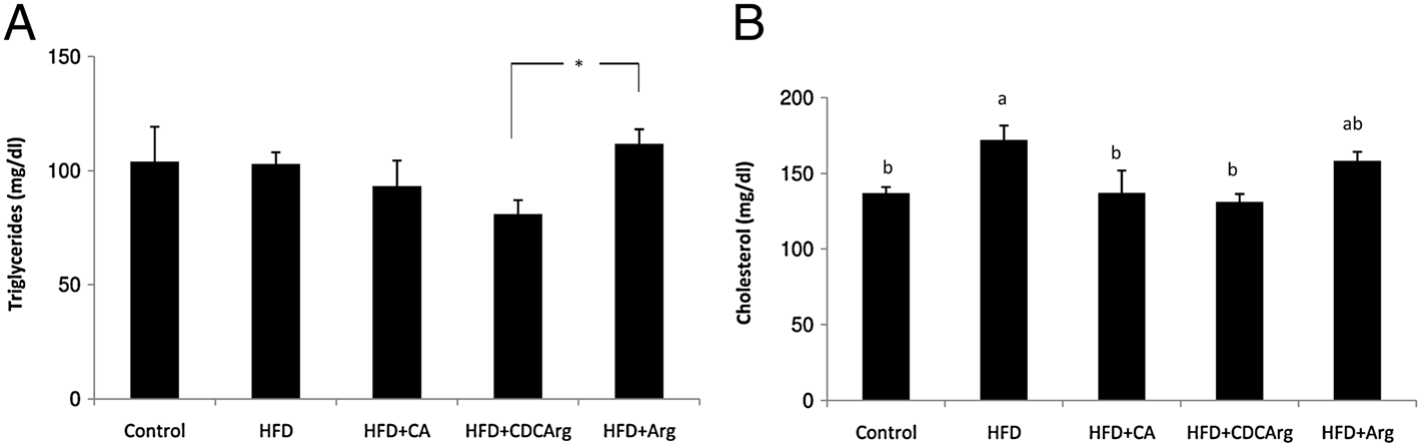
**Effect of CDCArg on serum lipids.** Mice were fed (ad libitum) for 5 weeks with LFD, HFD, HFD + CA, HFD + CDCArg and HFD + Arg. **(A)** Plasma triglycerides levels. **(B)** Plasma total cholesterol (total) levels. Means with different letters are statistically different, p < 0.05.

In HFD treated animals TG levels were evaluated. CDCArg had a tendency to ameliorate TG levels (Figure 5A). In addition, while plasma cholesterol levels were significantly elevated in HFD group no increase was evident in HFD + CDCArg and HFD + CA (Figure 5B).

### Effect of the compound CDCArg on FXR and SHP expression

**Figure 6:**
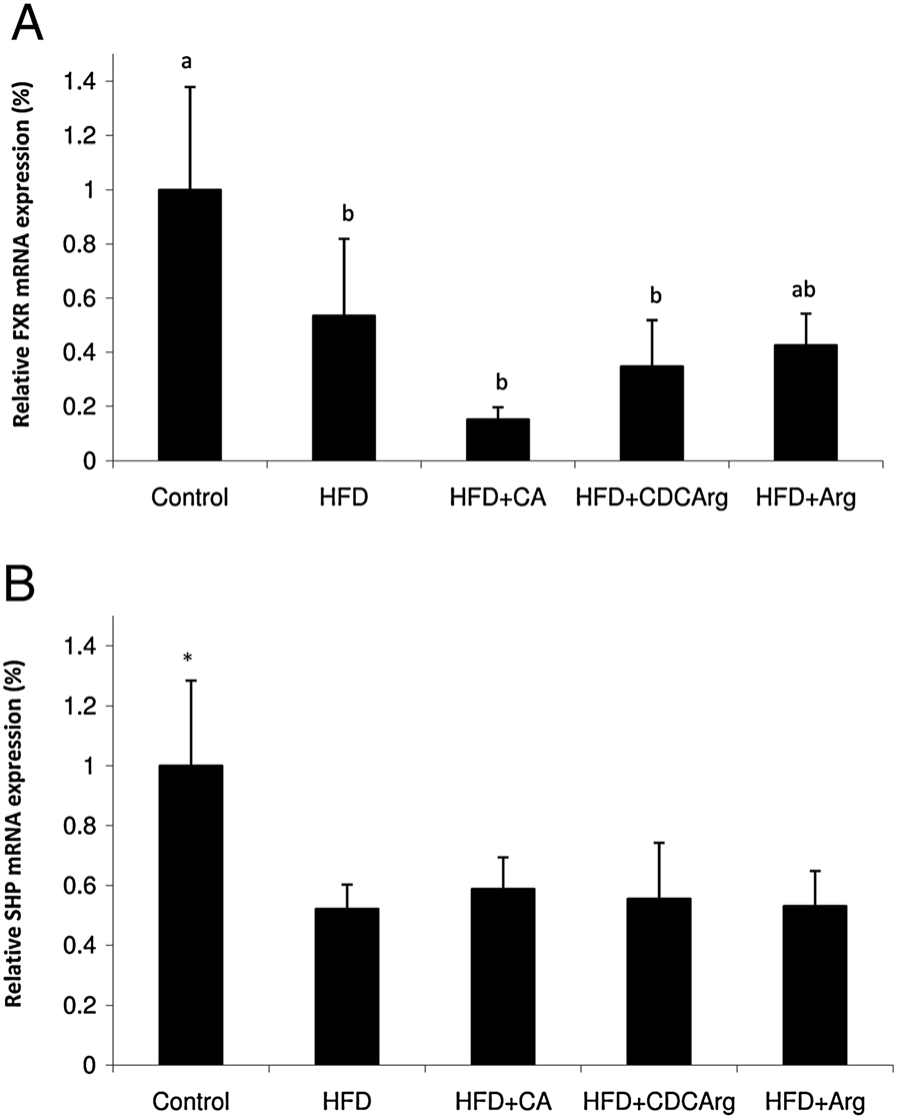
**FXR and SHP expression.** Mice were fed (ad libitum) for 5 weeks with LFD, HFD, HFD + CA, HFD + CDCArg and HFD + Arg. Gene expression of FXR and SHP were evaluated by RT-real time PCR, **(A)** FXR mRNA expression. Means with different letters are significantly different, LSD test P < 0.05. **(B)** SHP expression. Control is different from all other groups, Dunnett's test test p < 0.05. ribosomal 18S was used as the normalizing gene.

Since plausibly CDCArg can protect the liver by activating FXR nuclear receptor, the capacity of the compound to activate this orphan receptor was evaluated. FXR gene expression was suppressed by HFD and was further suppressed by the addition of CA (Figure 6A). Corresponding with the decrease in FXR expression, HFD also downregulated SHP mRNA levels, indicating limited capacity to activate FXR signaling pathways (Figure 6B). These results indicate that the FXR expression is suppressed by the HFD and even more by the addition of CA to the diet. In contrast, CDCArg did not further suppressed the expression of FXR as compared to HFD alone.

Recently, the accumulation of cholesterol was suggested to have pathological effects in the liver. Thus, in order to evaluate whether CDCArg has an impact on FXR activation in the presence of cholesterol, primary rat hepatocytes were incubated with cholesterol for 24 h with the addition of chenodeoxycholic acid (CDCA) or CDCArg. Under these conditions, only CDCA was able to activate FXR as was indicated by elevated mRNA levels of SHP (Additional file 2: Figure S2).

### Treatment study

In order to determine the effects of CDCArg following the onset of liver steatosis, mice were treated for 10 weeks with HFD (ad libitum) to induce hepatic fat accumulation. Thereafter, the HFD-fed mice were divided into two groups, with or without CDCArg 0.5% w/w. In this experiment, mice were pair-fed (15.5 kcal/day per mouse) to compensate the hyperphagic effect of CDCArg. The low fat diet (LFD) control group consumed on average the same amount of calories per day per mouse. The duration of treatment with CDCArg was 4 weeks.

### Effect of CDCArg on mice weight and metabolism

**Figure 7:**
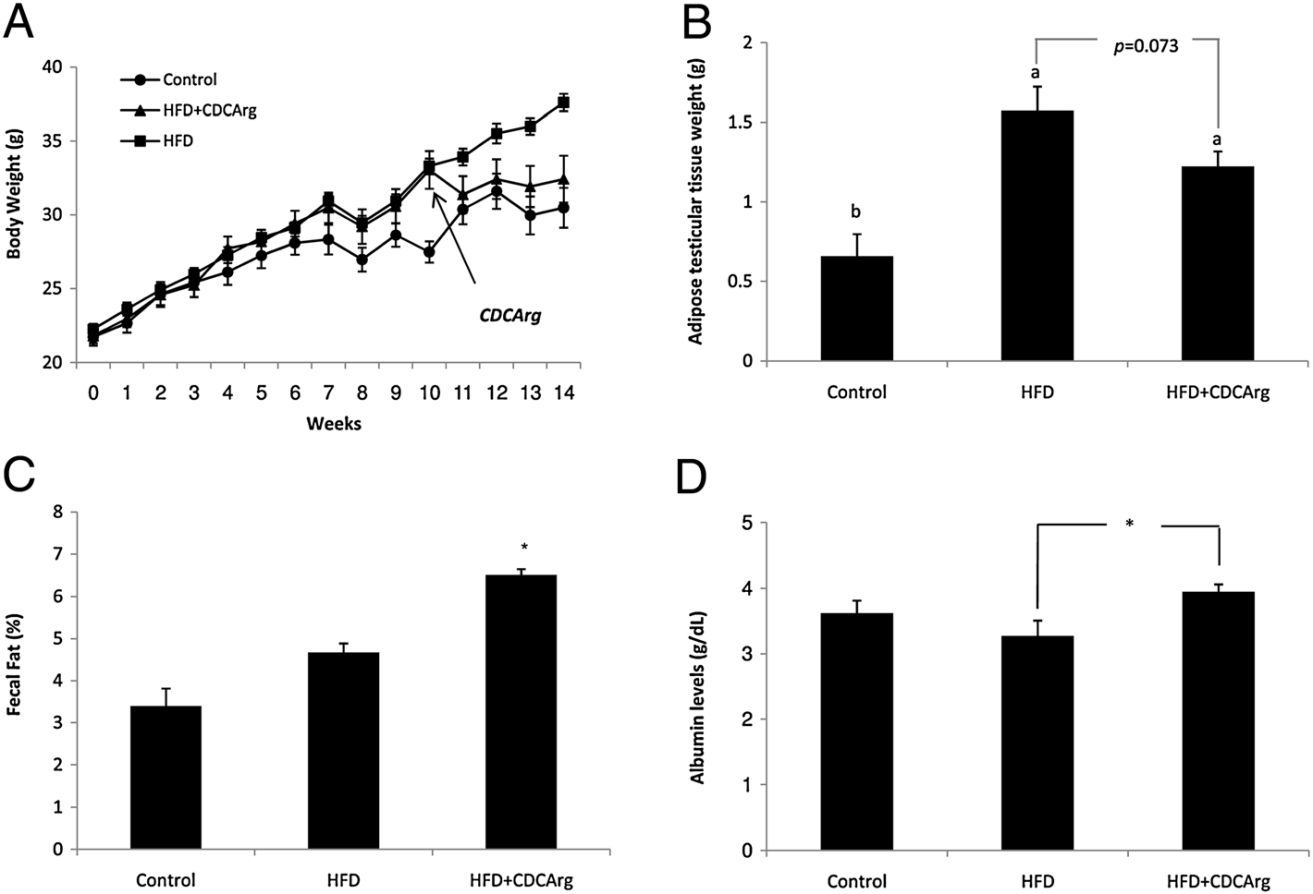
**Effect of CDCArg on mice weight, testicular adipose tissue weight, fecal fat and plasma serum albumin.** Treatments: Mice were fed with LFD for 14 weeks or with HFD for 10 weeks. At week 10 the HFD group was divided randomly into two equal groups that were pair fed. One group received HFD, while the other group received HFD + CDCArg. **(A)** Mice weight as measured during the experiment at the beginning of each week. *p < 0.05, lower weight compared to HFD. **(B)** Weight of adipose testicular tissue. **(C)** Feces fat percent (week 13): Total lipids were extracted from feces using Folch’s method after collecting feces during this week. Mean with *is statistically higher than HFD group, p < 0.05. n = 6, t-test. **(D)** Serum albumin levels in treated mice. *p < 0.05. t-test.

As expected, consumption of HFD for 10 week led to an increase in total body weight. However, while body weight further increased in mice continuing to consume HFD, the addition of CDCArg to HFD prevented continuation of weight gain (Figure 7A). Adipose tissue weight was also substantially lower in HFD + CDCArg group (Figure 7B). To assess the impact of CDCArg on intestinal fat absorption, fecal lipid contents were measured. As shown in Figure 7C fat content in feces was significantly higher in CDCArg treated group. Thus, CDCArg at least in part, inhibits intestinal fat absorption. According to the Merck veterinary manual: malabsorption syndrome in small animals and low plasma albumin levels may indicate lower protein absorption. Plasma albumin levels were significantly higher in HFD + CDCArg group than in HFD group, indicating that protein absorption was not compromised by the supplementation of CDCArg (Figure 7D).

**Figure 8:**
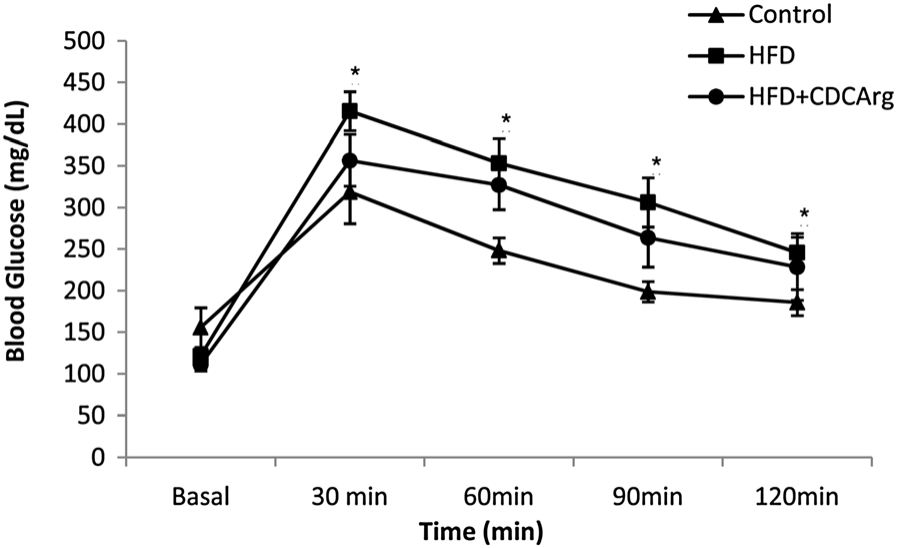
**Effect of CDCArg on insulin resistance as evaluated by intra-peritoneal glucose tolerance test (IPGTT).** IPGTT was perform at week fourteen. Values are expressed as mean ± SEM (n = 8-9 animals per group). Means with *are statistically higher, one way ANOVA and Post Hoc dunnett test.

The effect of CDCArg on insulin resistance was evaluated using interperitoneal glucose tolerance test (IPGTT) in mice. CDCArg supplemented mice exhibited a lower average peak in blood glucose during IPGTT, and improved glycemic response compared with HFD group (Figure 8).

### Effect of CDCArg in liver protection against HFD

**Figure 9:**
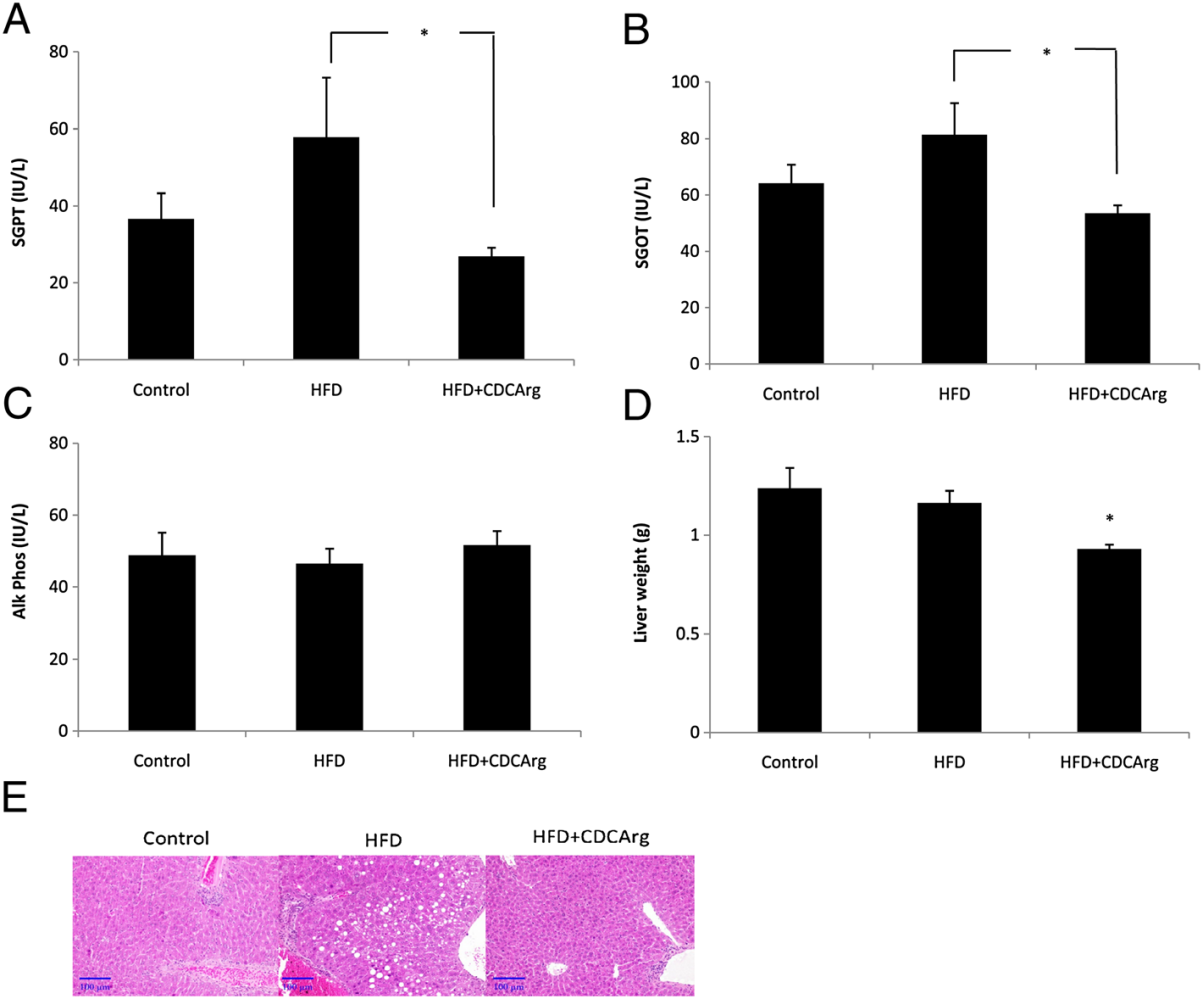
**Effect of CDCArg on serum hepatic enzyme levels, liver weight and liver histology.** Treatments: Mice were fed with LFD for 14 weeks or with HFD for 10 weeks. At week 10 the HFD group was divided randomly into two equal groups that were pair fed. One group received HFD, while the other group received HFD + CDCArg. **(A-C)** Average serum SGPT, SGOT or Alkaline Phosphatase levels. **(D)** Liver weight. **(E)** Representative liver histologic sections stained with H&E (Magnification 200X).

SGPT and SGOT levels were significantly lower in the HFD + CDCArg compared with the HFD group (Figure 9A,B). Alkaline phosphatase levels in plasma were not significantly altered (Figure 9C). Consistently, liver weights were also significantly lower in HFD + CDCArg group (Figure 9D). In agreement with these results, histological evaluation revealed lesser liver micro and macrovesicular steatosis in the HFD + CDCArg group than in the HFD group. Lobular inflammation was also observed in the control group but not in the HFD + CDCArg group (Figure 9E). Analysis of gene expression showed that CDCArg activates genes that are related to energy expenditure, peroxisome proliferator-activated receptor gamma coactivator 1-alpha (PGC1α) and peroxisome proliferator-activated receptor alpha (PPARα), while CDCArg suppressed the key gene that regulates denovo lipogenesis sterol regulatory element-binding protein 1c (SREBP1c) (Additional file 3: Figure S3.). No statistical difference in AMPK activation was observed in any of the treatments. However, the ratio of pAMPK to AMPK was tend to be higher in the HFD + CDCArg treated group (Data not shown).

### Effect of CDCArg in high cholesterol diet treated rats

In rats fed a high cholesterol diet, CDCArg unlike CA, did not potentiate the toxic effect of free cholesterol in the liver (Additional file 4: Figure S4). CDCArg did not aggravate the effect of cholesterol on liver mass or on the levels of hepatic transaminases in the serum.

## Discussion

The capacity of bile acids to protect or aggravate fatty liver disease is not clear. In terms of liver damage CA is considered a food toxin. Feeding rodents with bile acids has been used to study bile acid toxicity *in vivo*. The ability of the bile acids to produce hepatotoxicity is considered to be: UDCA < CA < CDCA < DCA < LCA [16]. Feeding rats a high cholesterol diet in the absence of bile acids resulted in relatively moderate liver damage compared to feeding of cholesterol in the presence of CA. In contrast to CA, CDCArg did not potentiate cholesterol toxicity (Additional file 4: Figure S4).

We have used bile acids in combination with saturated fatty acids from a plant source (palm stearin, containing around 50 percent of saturated fatty acids) that is cholesterol free. To the best of our knowledge, we are the first to report that CA administrated together with HFD rich in saturated fat in the absence of cholesterol potentiates liver damage. This was demonstrated by an elevation of liver enzymes in the blood. HFD, while contains fat of plants origin, has only a small capacity to induce liver damage. However, toxic bile acids may cause inflammation, apoptosis, and cell death [17]. Currently it is not clear if the deleterious effect of CA to the liver was synergistic or simply additive to the effect of the fatty acid rich diet. However, in contrast to CA, CDCArg did not generate toxic effects in the liver and did not induce general toxicity. Electrolyte imbalance and kidney damage were not observed during the *in vivo* treatments indicating no acute toxicity of the diets. CDCArg ameliorated HFD-induced liver steatosis. Also blood liver enzymes levels were lower normalizing the long term effect of feeding with HFD (treatment study). Hepatomegaly was corrected by CDCArg. All these parameters indicate that CDCArg may be used with efficacy and safety for NAFLD and NASH treatment.

In the clinical setting both CDCA and urosodeoxycholic acid (UDCA) have failed as an approved treatment for NASH [18]. The results of a randomized controlled trial of UDCA in NASH patients were disappointing, and no measurable benefit could be attributed to UDCA. Likewise, in a smaller, randomized, controlled study carried out in Switzerland no benefit of UDCA over placebo was found when looking at liver histology in NASH patients. However, there was a beneficial effect of UDCA on ALT levels in this trial.

Additionally, studies with another bile acid, the FXR activator obeticholic acid, that is under clinical investigation, have demonstrated adverse side effects of elevated LDL cholesterol [10].

Bile acids are used in an animal models of an atherogenic diet supplementation to potentiate the effect of cholesterol. It has been noted that the atherogenic diet can lead to the development of NASH. Atherogenic diets have been show to induce dyslipidemia, lipid peroxidation, stellate cell activation, precirrhotic steatohepatitis and cellular ballooning. Atherogenic diets in contrast to other animal models of NASH, such as the methionine and choline deficiency diet, induce the necessary histological features similar to those found in human NASH. It also generates a phenotype of insulin resistance. The addition of a high-fat component to the atherogenic diet exacerbated hepatic insulin resistance and further accelerated the pathology of steatohepatitis [12]. Therefore, it is extremely important to develop bile acid based therapy that is free of such deleterious effects.

Bile acid sequestrates have been used for many years to treat hypercholesterolemia and dyslipidemia. On the other hand, bile acid-activated nuclear and GPCR signaling pathways may protect against inflammation in the liver. In the current study, treatment with CDCArg improved fasting glucose levels, insulin and leptin sensitivity and reduced testicular fat accumulation induced by HFD in the mouse model.

Bile acids can enter the hepatocytes in several ways. The entry of bile acids into hepatocytes at the sinusoidal surface takes place by NTCP, Na^+^-dependent pathway that is driven by the transmembrane Na^+^-gradient [19]. NTCP is exclusively expressed in hepatocytes and localized at the sinusoidal membrane. In addition there is a less efficient system for uptake Na^+^-independent and involves several members of the organic anion transporting polypeptide family [19]. Another transporter, the apical sodium-dependent bile salt transporter is expressed at high levels in the terminal ileum, renal proximal tubules, and biliary epithelium. CDCArg, due to its net positive charge, most probably cannot or is not expected to be absorbed efficiently back into the liver and therefore is probably translocated into the colon dragging along fat and preventing fat absorption. However, evaluation of plasma albumin levels indicated that CDCArg did not impair protein absorption in mice.

In the current study we investigated a primary bile acid (chenodeoxycolic acid) that was conjugated to L-arginine. The arginine guanidino group of its side chain provides a constant net positive charge to the molecule throughout the range of physiological pH range. The pKa of guanidine is 12.5, indicating that this compound will exist almost entirely as a cation in the *in vivo* environment. According to our results such a bile acid did not potentiate cholesterol-induced liver damage and protected against deleterious effects of HFD composed of high amounts of saturated fat.

The effect of CDCArg in HFD-induced obesity in mice was tested. Two main effects were noted, increased consumption and decreased body weight gain. In general bile acids may possess an anti-obesity properties by activation of the TGR5 receptor. TGR5 is a novel pharmacological target in the metabolic syndrome and related disorders, such as diabetes, obesity, atherosclerosis, liver diseases and cancer [20]. However, its clinical effect is yet to be elucidated. Activation of TGR5 by bile acids releases incretins and improves glycemic control in a rodent nutritional model of HFD [21]. However, bile acids do not appear to be key mediators of the early increase in GLP-1 and PYY response in post-bariatric patients [22]. Therefore, the role of bile acids in obesity treatment is still unclear. Recently it has been shown that dietary fats, by promoting changes in host bile acid composition, can markedly alter conditions of the gut microbial environment due to sulfur containing bile acids, resulting in perturbation of immune homeostasis. A diet high in saturated fat promoted taurine conjugation of hepatic bile acids and promoted induction of colitis [23]. CDCArg, due to its different chemical properties, may offer a possible treatment for obesity as it directly affects weight gain. Its specific effects on gut polypeptide secretion and its anti-obesity mechanism still need to be clarified.

CDCArg + HFD in oppose to HFD decreased blood leptin levels, which could indicate the amelioration of obesity related leptin resistance. The effect of selective leptin resistance on disease progression and the metabolic syndrome is complicated [24,25]. Whether or not the effect of CDCArg is via leptin still needs to be clarified. CDCArg increased the expression of PGC1α and its related gene PPARα. This may accelerate mitochondrial oxidation of fat. Previously it has been reported that bile acids suppress PGC1 activity via the FXR and SHP pathway [26]. Since CDCArg is a different chemical structure compared natural bile acids, and was unable to activate the FXR pathway it does not suppose to suppress the PGC pathway and may regulate its expression by cAMP dependent pathway. Surprisingly, CDCArg also suppressed SREBP-1 expression probably in a mechanism that may be independent of SHP expression. All of these effects culminate in improvement of liver steatosis in the mice model.

In conclusion: conjugate of the bile acid chenodeoxycholic acid and the positively charged amino acid arginine was found to be not toxic in mice. The compound was evaluated as a liver protective agent in a model of NAFLD. These results indicate that the compound did not potentiate cholesterol accumulation in the liver and protected the liver from damage caused by a HFD rich in saturated fat. This compound was also effective in improving the metabolic condition of rodents treated with HFD and was found to be effective as anti-obesity agent in a mouse model of diet-induced obesity.

## Methods

### Animals and diets

Five-week-old C57BL/6 J male mice (purchased from Harlan Laboratories, Israel) were housed in a controlled environment (22–24°C, 60% humidity and 12 h light-12 h dark).

Experiments for prevention and treatment of NASH were performed. All bile acids and analogues were admixture into the diet. Fat source was palm stearin.

A) Prevention study- mice were randomly divided into five dietary groups (n = 8-9 each group). (1) Low-fat diet group (Control, LFD, 16% calories from fat). (2) High-fat diet group (HFD, 60% calories from fat). (3) HFD + CA (HFD + CA, 60% calories from fat + 0.5% w/w CA). (4) High-fat diet + chenodeoxycholyl-arginine ethyl ester (CDCArg) (HFD + CDCArg, 60% calories from fat + 0.5% w/w CDCArg). (5) High-fat diet + L-Arginine (HFD + Arg, 60% calories from fat + 1.25% w/v Arginine in drinking water). All mice had free access to food and water (ad libitum).

In our experiments we did not used CDCA as control to CDCArg due to it known hepatotoxicity. Therefore, CA which is the other primary bile acid was used.

B) Treatment study - mice were randomly divided into two dietary groups: (1) Low-fat diet group (Control, 16% kilo calories from fat, n = 5); (2) HFD (n = 16). After 10 weeks of dietary treatment, the HFD group was divided randomly into two equal groups (n = 8 in each group): (a) continued the previous HFD treatment (b) HFD + 0.5% w/w CDCArg. Food consumption and weight gain were evaluated and animals were adjusted to a pair fed diet of 15–16 k-calories/day/mouse.

#### *Rat study*

Since cholesterol has been designated recently to promote fatty liver disease and NASH [27,28], Therefore, male Sprague Dawley rats (8 per group) 160–200 gram were supplemented for 6 weeks with control diet (16% calories from fat), high cholesterol diet (HCD) (1%), HCD + 0.5% w/w CA, and HCD + 0.5% w/w CDCArg. Body weight was measured weekly and food intake was measured daily.

#### *Animal were sacrificed after an overnight fasting*

All animal procedures were approved by the local IACUC. Effect of Arginine-bile acid conjugats and cholesterol component of atherogenic diet on liver function AG-11-13096-2. Genomic and functional study of the fatty liver syndrome AG-10-12636-3.

### Chemical synthesis of CDCArg

A bile acid-arginine amino acid conjugate was prepared (Figure 1). The synthesized compound CDCArg was prepared and analytically characterized by Radikal therapeutics Inc, Herzelia, Israel (February 19, 2013) using LC-MS and NMR analysis. Purity of more than 95% was evaluated by HPLC- diode array detector.

### Liver histology

Animals were sacrificed and liver tissues were removed and fixed in 4% paraformaldehyde at room temperature. Liver sections were subjected to hematoxylin-eosin staining (**PathoVet** Veterinary Pathology Services, Rehovot, Israel).

### Blood parameters

Animal blood was taken from the inferior vena cava after anesthesia upon sacrificed. Blood glucose levels were measured using a handheld glucometer (Medisense). Analysis of plasma SGOT, SGPT, Alkaline Phosphatase, electrolytes (Na, Cl, K), creatinine and lipid profile (cholesterol and triglycerides) levels were performed by American Laboratories Ltd., Herzelia, Israel. Leptin and insulin levels were detected using a luminex assay kit (Millipore, Israel).

### Biochemical analysis

Total lipids were extracted from feces using Folch’s method [29] after collecting feces during weeks: 9–10 and 12–13.

### Intraperitoneal Glucose Tolerance Test (IPGTT)

Glucose tolerance was assessed using the intraperitoneal glucose tolerance test (IPGTT) in mice during the eighth and the fourteenth weeks of the study (experiment no.2). IPGTT was performed in 12 hours-fasted mice by injecting glucose (2 g/kg in 20% solution) intraperitoneally. Blood samples were obtained by cutting the tail tip and glucose concentration was measured after 0, 30, 60, 90, and 120 minutes.

### Analysis of gene expression

Genes which modulate by bile acids treatment were evaluated: Gene expression analyses were performed by RT and real time PCR using the following primers for mice and rats. SREBP1c was analyzed by western blot analysis and loading control was done by beta actin. Abs: Anti SREBP-1 Santa Cruse (Sc-367); Secondary goat Anti-Rabbit IgG, Jackson ImmunoResearch, Mouse Anti-Actin (612656) BD Transduction Laboratories. Secondary goat Anti-mouse IgG, Jackson ImmunoResearch.

### Mouse-

FXR r – 5'-TCA CTG CAC ATC CCA GAT CTC-3'

FXR f – 5'-TCC GGA CAT TCA ACC ATC AC-3'

SHP r – 5'-AGG ATC GTT CCC TTC AGG TA-3'

SHP f – 5'-CAG CGC TGC CTG GAG TCT-3'

18S f – 5'-ACC GCA GCT AGG AAT AAT GG-3'

18S r – 5'-CCT CAG TTC CGA AAA CCA AC-3'

PPAR-alpha f-5'-GTC ACA CAA TGC AAT TCG CTT T-3

PPAR-alpha r-5'-TTT GCT TTT TCA GAT CTT GGC A-3'

PGC-1 alpha f- 5'-AAA CCC TGC CAT TGT TAA G-3'

PGC-1 alpha r- 5'-TGA CAA ATG CTC TTC GCT TT-3'

GAPDH f - 5'-GCA TCT TGG GCT ACA CTG AG-3'

GAPDH f - 5'-AGA GTG GGA GTT GCT GTT GA-3'

### Rat-

SHP r – 5'-AGC CGT CGC TGA TCC TCA TG-3'

SHP f – 5'-ACT GCC TGT GCC AGC AAC AC-3'

18S f – 5'-CAC GGA CAG GAT TGA CAG AT-3'

18S r – 5'-CAA ATC GCT CCA CCA ACT AA-3'.

### Isolation and culturing of rat hepatocytes

Hepatocytes were isolated as follow: Briefly, after anesthesia, rat livers were perfused with Hanks’ balanced salt solution (HBSS) containing 1 mM EGTA, followed by perfusion with 0.05% collagenase (cat.LS004177, Worthington, Lakeswood, NJ) in HBSS in a recirculating matter. The liver was then detached and filtered through a 70 μm nylon mesh and cells were sedimented by centrifugation. Cells were plated onto six-well plates covered with fibronectin (700,000 cells/ml) and grown in low glucose Dulbecco’smodified Eagle’s medium (DMEM) supplemented with 10% fetal calf serum, 1% L-glutamine and 100 μg/ml streptomycin, when kept at 37°C in humidified atmosphere (95% air and 5% CO2) [30,31].

### Statistical analysis

Statistical analysis was performed using one way ANOVA and post HOC test **Fisher's** least significant difference test for experiments with multiple groups. Differences were considered significant at P < 0.05. The use of other statistical tests is mentioned in the figure legends.

## Abbreviations

NAFLD: Non-alcoholic fatty liver disease; NASH: Non-alcoholic steatohepatitis; HCC: Hepatocellular carcinoma; FXR: Farnesoid X receptor; SHP: Small hetrodimer partner; CA: Cholic acid; NTCP: Na^+^ /taurocholate cotransporter polypeptide; CYP7A1: 7 alpha-hydroxylase; CDCArg: L-Arginine ethyl ester and chenodeoxycholic acid conjugate; CDCA: Chenodeoxycholic acid; UDCA: Urosodeoxycholic acid; LFD: Low fat diet; HFD: High fat diet; TGR5: G protein-coupled bile acid receptor; SGPT ALT: Serum glutamic-pyruvic transaminase; SGOT: AST, Serum glutamic oxaloacetic transaminase; PGC1α: Proliferator-activated receptor gamma coactivator 1-alpha; PPARα: Peroxisome proliferator-activated receptor alpha; SREBP1c: Sterol regulatory element-binding protein 1c.

## Competing interests

The authors declare that there are no competing interests.

## Authors’ contributions

OT conceived the experimental design and performed the proofreading of manuscript. IV SA and MHO performed the experiments and the statistical analysis and wrote the manuscript. All authors discussed analyses and interpretation, read and approved the final manuscript.

## Supplementary Material

Additional file 1: Figure S1 Lack of toxic effects of CDCArg as indicated by electrolyte balance and renal function. Mice were fed (ad libitum) for 5 weeks with LFD, HFD, HFD+CA, HFD+CDCArg and HFD+Arg. **(A)** Serum creatinine levels. **(B-D)** Serum electrolytes (sodium, potassium and chloride, respectively) levels. Click here for file

Additional file 2: Figure S2 Expression of SHP following treatment with bile acids in isolated rat hepatocytes: Primary rat hepatocytes were isolated and seeded at concentration of 2 x10^6^ cells per well (in 6 well plates). Cells were treated with 0.05 mg/ml of cholesterol and with 100 μM of CDCArg or CDCA (n=3, p<0.05) for 18 h. Click here for file

Additional file 3: Figure S3 Effect of CDCArg on lipogenic gene expression and energy expenditure related genes in the liver. Treatments: Mice were fed with LFD for 14 weeks or with HFD for 10 weeks. At week 10 the HFD group was divided randomly into two equal groups that were pair fed. One group received HFD, while the other group received HFD+CDCArg. mRNA levels of **(A)** PGC1α, **(B)** PPARα. **(C)** Protein level of sterol regulatory element binding protein 1c (SREBP1). *p<0.05, statistically different from control by student t-test. GAPDH was used as housekeeping normalizing gene. Click here for file

Additional file 4: Figure S4 Effect of CDCArg and cholic acid (CA) in dietary cholesterol-induced liver damage. Rats were treated with LFD (control) or LFD + cholesterol (1% w/w) for 6 weeks in the presence and absence of bile acids (0.5% w/w). **(A)** Liver weight of animals after 6 weeks of diet treatments. Levels of two liver enzymes representing liver damage. **(B)** ALT (SGPT) and **(C)** AST (SGOT) levels. Means with different letters are statistically different, p<0.05. Click here for file
