# Supplementary figures and images for: L-arginine conjugates of bile acids-a possible treatment for non-alcoholic fatty liver disease
Source: Lipids Health Dis. 2014 Apr 22;13:69. doi: 10.1186/1476-511X-13-69 (PMC4021351; doi:10.1186/1476-511X-13-69)

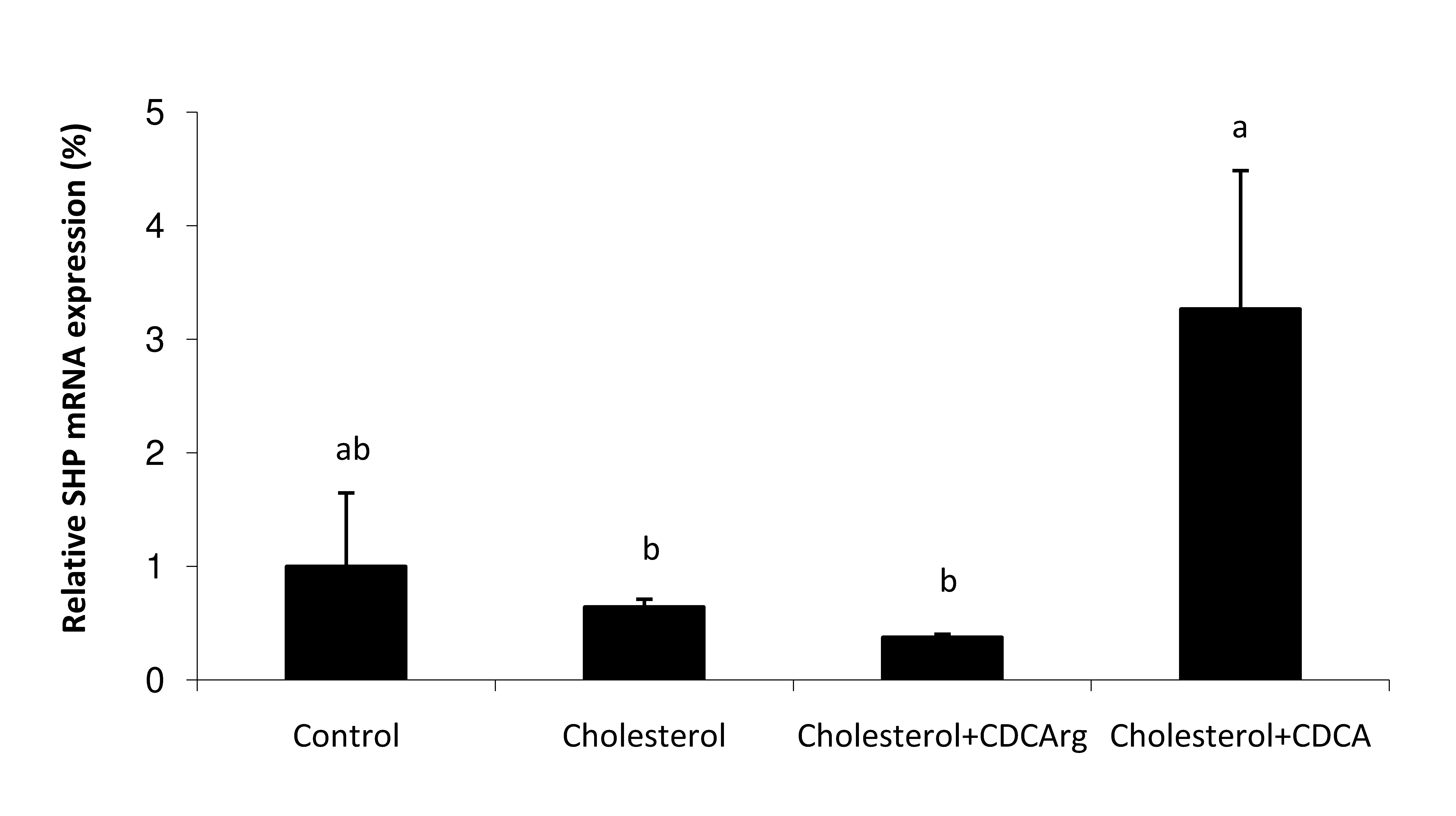

Supplement: Additional file 2: Figure S2 — Expression of SHP following treatment with bile acids in isolated rat hepatocytes: Primary rat hepatocytes were isolated and seeded at concentration of 2 x10^6^ cells per well (in 6 well plates). Cells were treated with 0.05 mg/ml of cholesterol and with 100 μM of CDCArg or CDCA (n=3, p<0.05) for 18 h. [file 1476-511X-13-69-S2.tiff]
